# The Role of Steroids in the Management of COVID-19 Infection

**DOI:** 10.7759/cureus.16841

**Published:** 2021-08-02

**Authors:** Zayar Lin, Wai Hnin Phyu, Zin Hnin Phyu, Tin Zar Mon

**Affiliations:** 1 Internal Medicine, California Institute of Behavioral Neurosciences & Psychology, Fairfield, USA; 2 Internal Medicine, University of Medicine 1, Yangon, MMR; 3 Internal Medicine, Sakura Hospital, Yangon, MMR

**Keywords:** coronavirus disease (covid-19), systemic steroids, covid-19 pneumonia, corticosteroids in covid-19, dexamethasone

## Abstract

Steroids are anti-inflammatory drugs that have been utilized in a wide range of clinical illnesses, including rheumatologic, autoimmune, inflammatory, and numerous lung diseases. Because of the inhibition of the inflammatory cascade, corticosteroids are beneficial in many pulmonary disorders, including asthma, chronic obstructive pulmonary disease (COPD), laryngotracheobronchitis, interstitial lung diseases, severe pneumonia, and acute respiratory distress syndrome. We will report a case of a COVID-19 patient treated with remdesivir, antibiotics, and steroids. We will also discuss the role of steroids in the management of COVID-19 patients.

## Introduction

Corticosteroids are hormones that are naturally produced from the adrenal cortex and are involved in a variety of physiological processes, such as inflammatory regulation, stress, and immunological response, protein, and carbohydrate metabolism. As a result, corticosteroids are critical in the management of autoimmune, allergic, malignant, and many inflammatory disorders [1].

In COVID-19-related severe acute respiratory syndrome, viral escape of cellular immune response and the cytokine storm is important in pathophysiology and clinical consequences. Dysregulation of cytokine and invasion of inflammatory myeloid cells results in lung inflammation and severe sequelae, such as acute respiratory distress syndrome, respiratory failure, sepsis, multi-organ failure, and death [2]. Corticosteroids have significant anti-inflammatory and anti-fibrotic effects, which may play a role in reducing pulmonary inflammation, especially in severe pneumonia and in advanced stages of COVID-19 disease [3]. However, the use of corticosteroids may reduce the immunological response, pathogen clearance, and promote viral replication, its downregulation effect may remain on the transcription of proinflammatory cytokines, consequently preventing the extensive cytokine response and promoting the resolution of pulmonary and systemic inflammation in pneumonia [4-6].

## Case presentation

**Figure 1:**
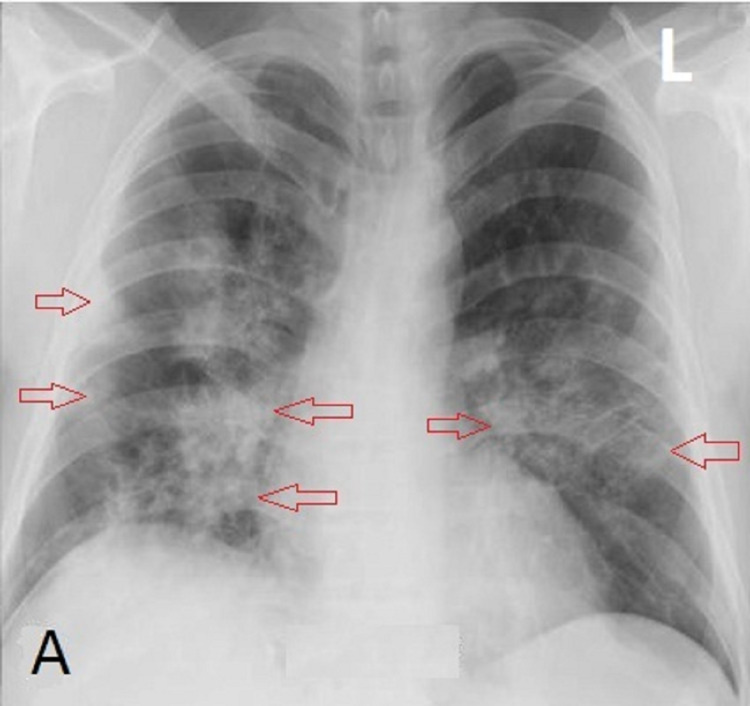
During admission, chest X-ray (A) showed bilateral ground-glass opacities (red arrows).

A 46-year-old man without underlying medical conditions came to the ED with shortness of breath, sore throat, and high fever for three days. At the ER, his oxygen saturation was low (around 82%), and his chest X-ray (CXR) showed bilateral ground-glass opacities with septal thickening in bilateral middle and lower zones and moderately severe pneumonia (Figure 1). He was given high-flow oxygen therapy (15 L), and his rapid antigen test for COVID-19 was positive. His COVID-19 reverse transcription-polymerase chain reaction (RT-PCR) test result also came back positive.

**Figure 2:**
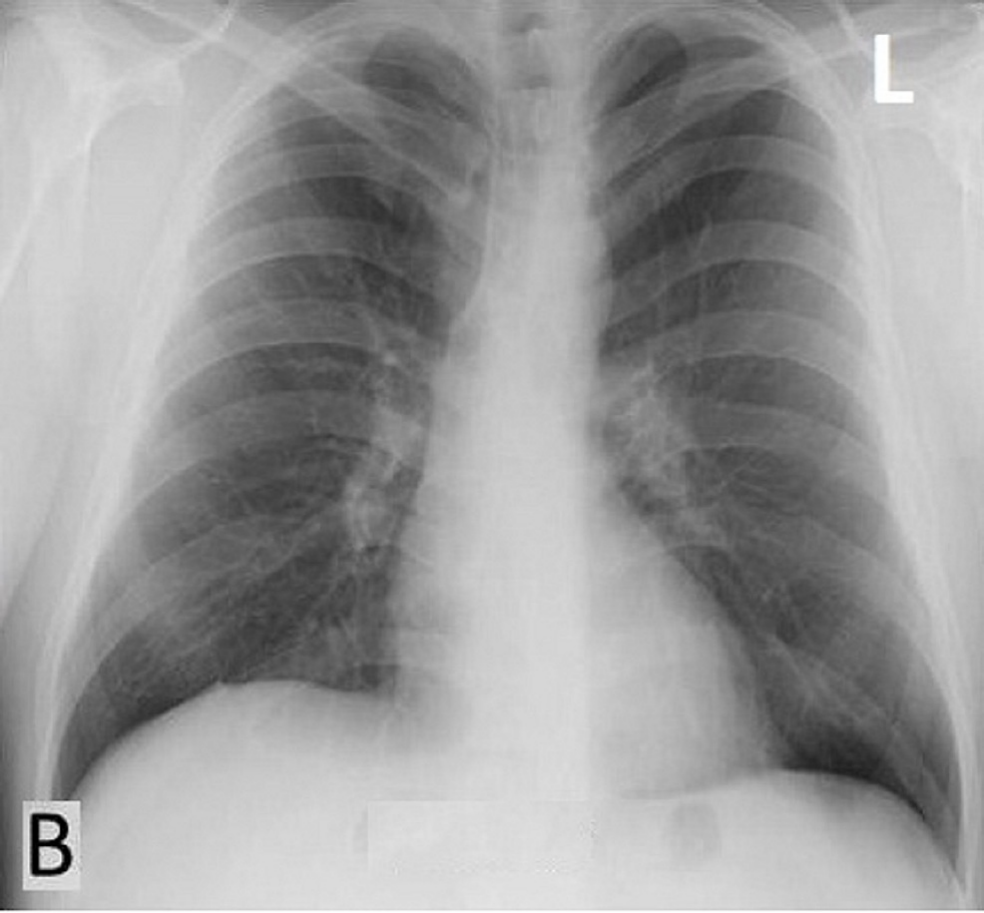
Follow-up chest X-ray (B) 30 days after onset of symptoms showed complete resolution of bilateral lung opacities.

His WBC count was slightly raised with lymphopenia and high C-reactive protein (CRP). He was given IV antibiotics (meropenem, cefepime, and moxifloxacin) with IV steroid therapy (low-dose dexamethasone 6 mg once a day). On day 2 after admission, he was given IV remdesivir once a day for five days. His D-dimer level was also increased, and he was given subcutaneous enoxaparin 0.4cc once a day for seven days. During the hospital stay, he suffered from hemoptysis and apathy. However, after receiving treatment for 10 days, his shortness of breath improved. His oxygen saturation returned to around 92-93% without oxygen therapy, and IV steroid was changed to oral medication with a tapering dose. The inflammatory parameters CRP and erythrocyte sedimentation rate (ESR) returned to the normal range after 14 days of treatment, and he was discharged on day 17. Repeated CXR was done on day 30 at the follow-up visit and witnessed complete radiographic resolution of lung opacities (Figure 2).

## Discussion

Corticosteroids are important hormones naturally produced by the adrenal glands in reaction to stress. They have effective anti-inflammatory and immunosuppressive properties related to the expression of proinflammatory genes via their glucocorticoid receptors. Because of these effects, corticosteroids play a crucial part in treating a large number of inflammatory conditions and autoimmune diseases, such as rheumatic arthritis, inflammatory bowel diseases, allergic conditions, chronic obstructive pulmonary disease (COPD), asthma, multiple sclerosis, hematological cancers, septic shock, and severe pneumonia. Acute pneumonia is an infection of the lungs that can be caused by viruses or bacteria and is often treated with effective antibiotics. Despite receiving proper antibiotic treatment, some cases of severe pneumonia result in serious complications, including death [7].

Corticosteroids have been proven to reduce cytokine releases, particularly interleukin-6 (IL-6) in serum and bronchoalveolar lavage in vivo, as well as CRP and neutrophil count in bronchoalveolar aspirates in people treated with corticosteroids [8]. 

Another advantage of corticosteroids in the treatment of pneumonia is that they prevent Jarisch-Herxheimer reaction to the administration of antibiotics in individuals with a high bacterial load. The Jarisch-Herxheimer reaction is hypothesized to be attributable to a high cytokine concentration immediately after initiation of antibiotics by the release of endotoxin or other bacterial mediators in patients with high bacterial load [9].

Severe community-acquired pneumonia (CAP) concerns tissue damage and organ failure caused by inflammation and is well-founded to reduce motility by the use of steroids [10, 11]. Several studies have shown the possibility of steroid treatment reducing overall mortality in severe CAP [12]. Eighteen patients with severe CAP need to be treated with steroids to prevent death. People with CAP who are treated with corticosteroids have reduced clinical failure rates, shorter hospital stays, and less morbidity [7].

A new coronavirus was identified in December 2019, as a result of the diagnosis of unusual pneumonia in Wuhan, China, and named as 2019 novel coronavirus (2019-nCoV) by WHO [13]. This is the third significant outbreak of coronavirus in the past two decades. Coronaviruses are RNA viruses that infect different organ systems of humans, livestock species, many mammals, and wild animals [14]. Corticosteroids, both oral and parenteral, have been utilized to manage serious respiratory symptoms in those patients infected with coronaviruses in the past [15].

The COVID-19-related severe acute respiratory syndrome is a syndrome of viral replication in combination with host immunological reaction [2]. The COVID-19 disease has an extremely high fatality rate due to respiratory failure combined with a significant cytokine storm, which is the host’s excessive immunological response to resist pathogen invasion. This type of accelerated immune response is thought to be responsible for the host tissue damage (pulmonary alveolar destruction and systemic inflammation), resulting in severe clinical outcomes (i.e., respiratory and multi-organ failure). Therefore, the management of COVID-19 infected patients is beneficial by targeting the host immune response and inflammatory cascade. Synthetic glucocorticoids, inexpensive, widely available, and simple to use, have been utilized in a wide variety of therapeutic settings, particularly for their immunosuppressive effects. Although there were no clear survival benefits, steroids have been used in treating COVID-19 patients with severe respiratory symptoms. Despite the fact that their immunosuppressive effects delay viral clearance, the use of steroids has been considered reasonable because of the resolution of respiratory failure being the most important concern in Covid-19 patients [16]. Furthermore, the new medical literature has revealed that the short-term use of methylprednisolone in the early stages of the disease can improve clinical outcomes and prevent disease progression [17].

## Conclusions

In this report, we presented a case of a COVID-19 infected patient with severe pneumonia. The patient was successfully treated and recovered without profound complications. On reviewing the treatment of the patient, we discovered that the role of steroids is still considered beneficial in the management of COVID-19 patients. The dose of steroids which is a low-dose dexamethasone 6 mg once/day, is fully supportive of recent medical literature. According to the results of a randomized trial, low-dose dexamethasone saves the lives of COVID-19 patients with severe pneumonia, reducing the chance of death by a third for those on ventilators and by a fifth for those on oxygen therapy. However, we still need further research studies to get stronger evidence in the near future.
